# Physicochemical Characterization and Evaluation of *Ficus vasta* Gum as a Binder in Tablet Formulation

**DOI:** 10.1155/2023/8852784

**Published:** 2023-08-09

**Authors:** Ashagrachew Tewabe Yayehrad, Tesfa Marew, Motlalepula Matsabisa, Gebremariam Birhanu Wondie

**Affiliations:** ^1^Department of Pharmaceutics and Social Pharmacy, School of Pharmacy, College of Health Sciences, Addis Ababa University, Addis Ababa, Ethiopia; ^2^Department of Pharmacy, School of Health Sciences, College of Medicine and Health Sciences, Bahir Dar University, Bahir Dar, Ethiopia; ^3^Department of Pharmacology, Faculty of Health Sciences, University of the Free State, Bloemfontein, South Africa

## Abstract

Binders are ingredients used in tablet granulation process for tablet cohesiveness which confirms that the tablet remains intact after compression. Natural gums have been employed as disintegrants, emulsifying agents, suspending agents, and binders in tablets. Even though *Ficus vasta* gum is claimed as a possible pharmaceutical excipient by some phytochemical studies, literature is scanty on its efficacy as a tablet binder. The purpose of this study was to isolate, characterize, and comparatively evaluate *Ficus vasta* gum as a potential binder in tablet formulation. Gum was extracted from *Ficus vasta* tree, characterized for physicochemical properties, and applied as a binder in paracetamol granule and tablet formulation. Granules were prepared using 4%, 6%, 8%, and 10% w/w concentration of the gum and standard binders (polyvinylpyrrolidone K-30 and Starch^@^1500) by wet granulation. The formulated tablets were then evaluated for tablet quality parameters, and comparison between the test and standard binders was done by ANOVA. The dried crude gum yielded 50.63% (w/w) of a brownish yellow purified gum. The angle of repose, Carr's index, and the Hausner ratio all complied with the pharmacopoeial recommendations. The gum is compatible with the model drug, paracetamol. The paracetamol granules prepared with *Ficus* gum binder demonstrated an optimum size range and size distribution with substantial flow and compressibility properties. *Ficus* gum binder demonstrated significantly higher disintegration time and strength properties than that of similar concentrations of Starch^@^1500 but lower than polyvinylpyrrolidone (*p* < 0.05). *Ficus* gum has better binding properties than starch but lower than polyvinylpyrrolidone. Hence, *Ficus vasta* gum can be used as an alternative tablet binder in tablet manufacturing.

## 1. Background

In the pharmaceutical industry, the excipient is a catch-all term that includes various subgroups comprising diluents or fillers, binders or adhesives, disintegrants, lubricants, glidants, flavorants, colorants, and sweeteners [1]. They are included to impart stability; ensure accuracy, precision, and homogenous blending; mask a bitter taste; improve flowability; add bulk density; and control the release thereby improving patient compliance, bioavailability, efficacy, and reducing toxicity [2]. Excipients are manufactured through a significant chemical change, physical modification, blending, or purification which causes many of the other components present in the starting materials to be removed or reduced [3]. New and modified excipients continue to emerge with better drug delivery performance, but of particular interest is the increasing trend in research into plants [4]. Plant-based excipients have diverse applications as a diluent, binder, disintegrant, thickeners, gelling agents, and bases [5]. The extraction, synthesis, and characterization of natural excipients can aid the drug development and delivery design to achieve the desired set of performance standards [6].

Binders are the ingredients used in the tablet granulation process for tablet cohesiveness which confirms that the tablet remains intact after compression [7]. They are dry powders or liquids, which are added during granulation to promote the granulation process, to promote cohesive compact during direct compression, and to provide mechanical strength to the tablet [8–10]. Synthetic binders are special paving materials manufactured by mixing polymers, resins, and oils, but they have certain drawbacks such as high cost, non-renewable sources, side effects, toxicity, causing environmental pollution during their synthesis, and nonbiodegradable (whereas biodegradable synthetic polymers are costlier) [11]. Natural binders like different starches, gums, and mucilages possess binding capacity as well as some other properties like disintegrant, filler, and sustained release being much safer and more economical than synthetic polymers [7]. These are widely applied due to their low toxicity, biodegradability, availability, and low cost. They are also applied in the formulation of modified-release dosage forms by modifying drug release patterns, and the bioavailability of the drug [8].

Granules are particle aggregates of mixed ingredient powders that are bonded to each other by the addition of a binder component for enhanced strength properties [12]. Both the physicochemical properties and the concentration of the binder in the blends influence the mechanical properties of granules [13]. A cohesive linkage between each ingredient is expected to be generated by the incorporation of the binder, which can result in the production of granules and tablets with preset quality standards [14]. Binder is considered to be the most fundamental factor in determining the granule properties and quality of the tablet [15]. Generally, tablet tensile strength differs based on the binder type and process parameters [16]. The moisture-retaining capacity of granules greatly depends on the type and concentration of the binding agents [17].

Natural gums have been investigated and employed as disintegrants, emulsifying agents, suspending agents, and binders in immediate and sustained release preparations [4]. Gums are known for their relative inertness, ready availability, and cheapness. They are long, straight, or branched chain polysaccharides with hydroxyl groups that can be bonded to water molecules [9]. There are some commonly known and commercially available natural gums used as binders such as gum acacia or gum arabic, gum karaya, gum tragacanth, and xanthan gum [8]. These are generally believed to show good potency as binding agents, but there are continuous efforts to have more marketable natural binders from plant gums [18]. Some of the natural gums that demonstrated comparable binding capacity with standard binders like PVP and acacia include Grewia gum of *Grewia mollis* [19], almond gum of *Prunus amygdalus* [20], cashew gum of *Anacardium occidentale* [21], *Aegle marmelos* (Cordia) fruit gum [22], *Mangifera indica* gum [23], and gum from *C. olitorious* dried leaves [9]. In addition, gum of cederela from *Cedrela odorata foliage*, [24], kondagogu gum and ghatti gum from *Brachystegia Eurycoma*, [25], gum kondagogu of *Cochlospermumgossypium*, [26], and gum tamarind of *Tamarindus indica*, [27] demonstrated good tablet binding properties with different promising physicochemical characteristics. Gum exudates of *Terminalia randii* also resulted in tablets with an increased crushing strength friability ratio (CS/FR) which is one quality parameter for oral tablets [28].

*Ficus* is a genus of plant family *Moraceae* that includes about 850 species. Most of these species are edible and have nutritional importance for humans. It has a gummy latex-like material within its vasculatures that provide protection and wound healing from physical assaults [29]. A common characteristic of latex produced by different plants is its advantage as one source of natural gums [30]. Most *Ficus* plants, commonly known as “fig trees” have been used for human consumption for centuries, and recently, their nutritive, pharmaceutical, and pharmacological values have been investigated [31]. *Ficus vasta* Forssk, locally known as “warka” in Ethiopia, is a multipurpose tree found in various parts of dry north and eastern Africa, Sudan, Ethiopia, Somalia, Saudi Arabia, Uganda, Tanzania, along rivers, and in dry savannah [32, 33]. In Ethiopia, mature trees of *F. vasta* are seen along roads, riverine areas, in farmlands, lowlands, rocky landscapes, as well as in dry savannah, and within the Rift Valley [34]. Ethnobotanical studies in Ethiopia also revealed the multidimensional importance of *Ficus vasta* Forssk [35, 36]. *Ficus vasta*, the plant found in Ethiopia and some neighboring countries, is the 10^th^ of the 30 most abundant species (2.3%) in Ethiopian forest coverage, which may be an abundant source for pharmaceutical industries [37]. The fruits of *Ficus* tree are edible in most parts of Ethiopia. There is also a traditional practice of preparing chewing gum directly from the latex in the community. This may be a temporary assurance for its safety. But, further investigations are required in this regard. The plant can be cultivated widely in dry and uncovered areas [35, 36, 38].

Ethiopia is one of the major producers and exporters of natural gums from different indigenous tree species. Over 60 gum and resin-bearing species are found in the country [39]. Tadesse et al. [38] investigated the Ethiopian species of *Ficus* (*F. vasta* Forssk) and concluded that it contains gum. Several studies indicated that different species of *Ficus* demonstrated different amounts of gum yield [31, 40, 41]. Ahad et al. [11] investigated the binding nature of one of the *Ficus* specious, *Ficus reticulate*, and finally concluded that it can be used as a binder in pharmaceutical dosage forms. Hence, investigating the potential application of this *Ficus* gum as an excipient will be a possible addition to such indigenous production and export profiles of the country. In the present study, isolation and purification of the bark-incised exudate of *F. vasta* gum were done, and the binding ability of the gum in tablet formulation compared with a standard binding agent was determined.

## 2. Materials and Methods

### 2.1. Materials

Local *warka* tree gum was collected from Bahir Dar (Ethiopia). Paracetamol USP working standard was obtained from EFDA (Addis Ababa, Ethiopia). Paracetamol IP with other necessary tablet excipients, i.e., Starch^@^1500 (Guangdong Guanghua Sci-Tech Co. Ltd, China), polyvinylpyrrolidone (Guangdong Guanghua Sci-Tech Co., Ltd, China), lactose (Narula Exports Pvt. Ltd., New Delhi, India), talc (Narula Exports Pvt. Ltd., New Delhi, India), and magnesium stearate (Narula Exports Pvt. Ltd., New Delhi, India) were obtained from Ethiopian Pharmaceutical Manufacturing Company S.C (EPHARM). Muslin cloth, ethanol, NaOH pellets (Guangdong Guanghua Sci-Tech Co., Ltd, China), potassium dihydro orthophosphate (Guangdong Guanghua Sci-Tech Co., Ltd, China), acetone (Labmark Chemicals Pvt. Ltd., India), and chloroform (Sisco Research Laboratories Pvt. Ltd, India) were purchased from the market.

### 2.2. Methods

#### 2.2.1. Gum Extraction and Purification

The gum exudate of *Ficus vasta* (*warka*) was collected in May 2021 from Bahir Dar City, specifically from around the Abay river basin. Extraction and purification were done following the methods described by Choudhary and Pawar [42] and Nep et al. [43], respectively, with some modifications. The gum was collected by making an incision on the bark of the *Ficus vasta* tree. The collected gum was dried with a tray oven drier (KEMI Model: KOA.6.F, INDIA) at 50°C for 6 hours. The dried gum powder was pulverized using a mortar and pestle, sieved by a No. 60 (250 *μ*m) sieve (SETHI Standard Test Sieve, IS: 460, India), and then, a kilogram of the gum powder was dissolved in 5 liters of distilled water. After filtration with a muslin cloth to separate insoluble residues, the gum solution was then precipitated using 97% ethanol (1 : 2). The precipitate was dried with a tray oven drier at 50°C for 6 hours and powdered with mortar and pestle. For the purification purpose, the gum extract was sieved by a No. 60 (250 *μ*m) sieve (SETHI Standard Test Sieve, IS: 460, India), solubilized using distilled water, then refiltered, and reprecipitated by 97% ethanol (1 : 2). The purified gum was finally oven-dried, powdered, and kept in a closed container for characterization and further use as a binder.

#### 2.2.2. Gum Characterization

*(1) Physicochemical Characteristics of the Gum*. *Percentage Yield:* The purified gum powder was weighed and divided by the weight of the crude gum which is directly dried after collection to obtain the percentage yield (equation (1)). For comparison of the yield potential, *Ficus* trees of different age were considered. The latex was also directly diluted and precipitated to estimate the percentage yield in w/v (equation (2)) [44, 45].

(1) $$\begin{array}{c} \%\text{Yield}=\frac{\text{Purified weight}}{\text{Crude weight}}\times 100, \end{array}$$

(2) $$\begin{array}{c} \%\text{Yield}=\frac{\text{Purified weight}}{\text{Crude latex volume}}\times 100. \end{array}$$

*Ash Value:* The percentage ash content was determined according to the BP procedures [46]. Accordingly, a mucilage of the purified *Ficus* gum was prepared, and 2 g of the mucilage was weighed, evenly distributed in the crucible, and dried at a temperature of 105°C for one hour. Then, it was ignited in a muffle furnace (CARBOLITE, OAF 11/1, England) at 450°C for 8 hours. The percentage ash was then calculated using equation (3).

(3) $$\begin{array}{c} \%\text{Ash value}=\frac{\text{CR}-E}{C-E}\times 100, \end{array}$$

where *E* is the tarred weight of crucible, CR is the weight of crucible + residue, and *C* is the weight of crucible + test portion.

*Presence of Starch:* 10 ml of *Ficus* gum solution (10% w/v) was boiled and cooled. Then, 0.1 ml iodine test solution was added to check for the presence of starch or dextrin [46].

pH: pH meter (HANNA Instruments Ltd, Romania, Europe) was used to determine the pH of a 1% w/v dispersion of the gum powder in distilled water [47].

*Densities and Density-Related Properties:* The bulk density was determined by transferring 30 g of gum powder into a 250 ml measuring cylinder. The volume occupied by the powder was read, and bulk density was calculated as g/ml. The bulk in the cylinder was then tapped 500 times for 4 min using a tapped densitometer (ERWEKA, Type SVM, Germany). The volume occupied was used to calculate tapped density as g/ml. Carr's index and the Hausner ratio were then calculated with equations (4) and (5), respectively [48].

(4) $$\begin{array}{c} \text{Car}{\text{r}}^{\prime }\text{s index }\left(\%\right)=\frac{\text{Tapped density}-\text{bulk density}}{\text{Tapped density}}\times 100, \end{array}$$

(5) $$\begin{array}{c} \text{Hausne}{\text{r}}^{\prime }\text{s ratio}=\frac{\text{Tapped density}}{\text{Bulk density}}. \end{array}$$

*Flow Rate and Angle of Repose:* The flow rate and angle of repose were determined by the funnel method [48]. 30 g powder was allowed to flow through a stemless funnel having a 15 mm aperture from a 10 cm height. Time (sec) for the duration of flow and the average diameter and height of the powder piles formed were recorded. The flow rate and angle of repose were then determined from the recorded data using equations (6) and (7), respectively.

(6) $$\begin{array}{c} \text{Flow rate}=\frac{\text{mass}\left(\text{g}\right)}{\text{time}\left(\sec\right)}, \end{array}$$

(7) $$\begin{array}{c} \text{Angle of repose }\left(\theta \right)=\tan^{-1}\left(\frac{h}{r}\right). \end{array}$$

*Swelling Power (SP) and Solubility Index (WSI):* These were determined as per BP procedures [46]. Firstly, 0.5 g gum powder was weighed directly into preweighed centrifuge tubes, and 10 ml of distilled water was added to each tube. The tubes were then kept in a thermostatically controlled water bath (HH-S4 Digital Thermostatic Water Bath, XMTE-205, China) at 25°C for 30 min with frequent mixing at 2 min intervals. The tubes were then cooled and centrifuged (Labtech AC-2304 centrifuge, Labtech International Ltd, Indonesia) at 3000 rpm for 15 min. The sediment weight was recorded while the removed supernatant was dried to constant weight in an oven at 105°C for 12 hrs. Then, WSI and SP were calculated using equations (8) and (9), respectively.

(8) $$\begin{array}{c} \text{WSI}=\frac{\text{Dried supernatant weight}}{\text{sample weight}}\times 100, \end{array}$$

(9) $$\begin{array}{c} \text{SP}=\frac{\text{Sediment weight}}{\text{Sample weight }\left(100-\text{WSI}\right)}\times 100. \end{array}$$

*Relative Solubility*: The relative solubility was determined in cold and hot distilled water, acetone, chloroform, and ethanol. Accordingly, 1 g of gum was added to 10 ml of each solvent and left overnight. Five ml of the clear supernatants was taken in small preweighed evaporating dishes and heated to dryness over a digital thermostatic water bath at 50°C for organic solvents and at 105°C for distilled water for 2 h. Triplicate measurements were made. The ratio of the dried soluble mass (SM) to the volume of sample solution (VS) was determined as its percentage solubility using equation (10) [49].

(10) $$\begin{array}{c} \text{Solubility}=\frac{\text{SM}}{\text{VS}}\times 100. \end{array}$$

*Viscosity Studies:* Different concentrations (4.0, 6.0, 8.0, and 10% w/v) of each of the *Ficus* gum, PVP, and starch were prepared, and viscosity was measured with a viscometer (BROOKFIELD CAP 2000+ viscometer) at room temperature with spindle number 3 [28].

*Loss on Drying:* Five gram of gum powder was dried at 100 ± 5°C till the constant weight was obtained, and then, the percentage loss on drying (%LOD) was determined from the initial weight (*w*1) and weight after drying (*w*2) using equation (11) [22]. Triplicate measurements were made.

(11) $$\begin{array}{c} \%\text{LOD}=\frac{w1-w2}{w1}\times 100. \end{array}$$

*Moisture Sorption-Desorption Studies:* A 2 g of gum powder was weighed onto the dry preweighed evaporating dish. The final weight of the dish was noted and then placed over water in a desiccator for 5 days, thereafter removed and transferred into another desiccator over a desiccant for another 5 days. The dish with its content was weighed daily [49].

*Particle Nature:* The crystallinity nature of the gum extract was analyzed by X-ray diffraction analysis using an XRD-7000 X-ray diffractometer (MAXima, SHIMADZU Corporation, Japan) at 40 KV and 15 mA with a scanning diffraction angle range of 10 to 60°C 2-theta. Percentage crystallinity was determined from the XRD data by calculating the area of peaks using OriginPro2022® (OriginLab Corporation, MA, USA) [49].

*Compatibility Studies:* The drug-excipient compatibility study was conducted by instrumental analysis using Fourier transform infrared spectrophotometry (ATR-FTIR, Tensor II, Bruker, Germany) and Differential Scanning Calorimetry (PerkinElmer DSC 4000, USA). The pure drug, the gum, and their 1 : 1 physical mixture were examined during the analysis [43].

#### 2.2.3. Preparation and Evaluation of Granules

*(1) Granule Preparation*. All the granulation process was done using starch, lactose, talc, and magnesium stearate as a disintegrant, filler, glidant, and lubricant, respectively. Different concentrations (4, 6, 8, and 10% w/w), as decided after a preliminary investigation, of each of the *Ficus* gum, PVP K-30, and Starch^@^1500 were added as binders using the quantities stated in Table 1. A tubular mixer (Willy A. Bachofen AG, Turbula 2TF, Basel, Switzerland) was used to form a primary powder mix of the drug, the diluent, and the binders. For the Starch^@^1500 binder, the starch paste was prepared and added to the dry mix. The disintegrant (starch) was added portion by portion (half before granulation and half after granulation). Then, distilled water was added as granulating liquid, and the final mass was subjected to pass through a 1.6 mm sieve (ERWEKA, Type AR 401, Germany). The wet mass was then transferred to a Petri dish and dried in an oven (Kottermann® 2711, Germany) at 105°C for 30 minutes. Finally, the dried granules were mixed using the turbular mixer with the rest of the ingredients: magnesium stearate, talc, and the disintegrant starch mass and passed through a 1 mm sieve (ERWEKA, Type AR 401, Germany) [49].

*(2) Granule Evaluation*. *Granule Flow & Compressibility:* The characterization of granules based on physicochemical properties such as densities, flowability, and compressibility was carried out according to the standard procedures used for the *Ficus* gum characterization [9].

*Granule Size Distribution & Mean Granule Size:* Mean size and size distribution of granules were also determined by using a sieve method. Thirty grams of granules from each batch was put in a set of sieves (ERWEKA, Type AR 401, Germany) arranged in mesh size from top to bottom with the widest sieve on the top (sieve size order of 1000 *μ*m, 710 *μ*m, 315 *μ*m, and 224 *μ*m). After shaking the seives for 2 minutes, the granules remaining on each sieve were weighed, and percent granules retained on each sieve were recorded. From this data record, the granule size distribution (GSD) and the mean granule size (MGS) were calculated for each formulation using equations (12) and (13), respectively [50].

(12) $$\begin{array}{c} \text{GSD}=\frac{\text{Retained mass}}{30\text{g}}\times 100, \end{array}$$

(13) $$\begin{array}{c} \text{MGS}=\frac{\sum \left(A\text{verage consecutive sieve size}\times \text{MGS}\right)}{100}. \end{array}$$

*Granule Friability:* Ten gram (*W*1) of each granule formulation with a granule size greater than 315 *μ*m was placed into the friabilator (ERWEKA TAR 20, Germany) and allowed to spin at 25 rpm for 4 minutes. The granules were then sieved again with a 315 *μ*m sieve. The retained granules were reweighed (*W*2), and the percent loss was calculated as granule friability with equation (14) [46]. Triplicate measurements were made.

(14) $$\begin{array}{c} \%\text{Loss}\left(\text{friability}\right)=\frac{W1-W2}{W1}\times 100. \end{array}$$

#### 2.2.4. Tablet Formulation and Quality Evaluation

The prepared granules and all the necessary ingredients were compressed into flat tablets using a tablet compression machine (Eco Press 200, Parle Elizabeth Tools Pvt. Ltd, India) and then evaluated for the following listed mechanical and drug release properties based on the methods approached by Desta et al. [49] and Tahir et al. [51].

Weight Variation: Twenty tablets were picked randomly for each batch and weighed individually to calculate the mean weight with standard deviation.

Friability: 20 tablets were selected randomly and weighed together using the electronic balance and then placed in a friabilator (FTA-023, Single Drum, India). The machine was operated for 4 min, at 25 rpm. Finally, the tablets were taken out from the friability tester, dedusted, and weighed once again. The percentage losses were determined as % friability (w/w).

Thickness, Diameter, and Hardness: Five tablets were selected at random from each batch to perform these tests using a combined thickness-diameter-hardness tester (Sotax HT, Model: HT 1,500 N, Switzerland). A mean value was then calculated for each batch with their standard deviations. The tensile strengths (*T*) of the tablets were determined from the diameter, hardness, and thickness data of tablets by applying equation (15).

(15) $$\begin{array}{c} \text{T}=\frac{2F}{\pi dt\;}, \end{array}$$

where *T* is the tensile strength of the tablet (kg/cm^2^), *F* is the load (MN) needed to cause a fracture, *d* is the tablet diameter (m) and *t* is the thickness (m).

Disintegration: Five tablets were selected at random from each batch and placed in each of the cylindrical tubes of the disintegration apparatus (ERWEKA, Germany). The time required for the individual tablet to breakdown into fine particles and pass out through the mesh was recorded. The mean disintegration time was calculated for each batch.

Dissolution rate: The USP type II dissolution apparatus (ERWEKA, Germany) was used for the *in vitro* drug release study. Six randomly selected tablets from each batch were assayed putting one tablet per the vessel of the dissolution apparatus. Then, the placed tablet was subjected to a paddle rotation speed of 50 rpm for a predefined period in 900 ml phosphate buffer medium with a pH of 5.8 at 37°C which is set based on the pharmacopoeial recommendations of dissolution test standard for acetaminophen. A dissolution profile was recorded by taking a 1 ml sample from the dissolution medium at 5, 10, 15, 30, 45, and 60 minutes. An equal volume of fresh dissolution medium was replaced for each sample withdrawn. The samples withdrawn were filtered using Whatman filtration equipment (0.45 *μ*m PVDF W/GMF) and diluted using the medium to obey the Beer-Lambert Law. Then, the UV absorbance was measured using UV-Vis spectroscopy (ERWEKA, T80+ Spectrophotometry, Double Beam, Ver 3.3, PG Instruments Ltd) at 243 nm.

From the spectrophotometric data, the rate of drug release was calculated using the formula obtained from the calibration curve. For constructing the calibration curve, a stock solution containing 0.2 mg/ml of paracetamol in phosphate buffer of pH 5.8 was prepared and diluted to five different concentrations (4, 6, 8, 10, and 12 *μ*g/ml). Phosphate buffer was used as a blank. The regression equation from the calibration curve was applied to convert the absorbance into concentration at each time point of sampling. Then, percentage cumulative release was used to characterize and compare the *in vitro* drug release profile of the tablets at each binder concentration.

#### 2.2.5. Statistics

Data registration and statistical analysis were done using Microsoft Excel 2010, IBM SPSS Statistics 26, and OriginPro2022® (OriginLab Corporation, MA, USA). OriginPro was also used to draw figures showing the results. Comparative analysis of the effects of binders on granule and tablet properties was performed with one-way analysis of variance (ANOVA) assisted with Tukey's post hoc analysis at a 95% confidence interval (*a* = 0.05). Observed *p* values ≤ 0.05 were considered significant. Triplicate measurements were done necessarily, and the values are given as mean and standard deviation (mean ± SD).

## 3. Result

### 3.1. Physicochemical Characteristics of the Gum

#### 3.1.1. Physicochemical Properties of *Ficus* Gum

The dried crude gum yielded 50.63 ± 1.15% (w/w) of a brownish yellow-colored purified *Ficus* gum with a pH of 7.62. The water solubility index was demonstrated to be 62.6%. The gum is freely soluble in water, while sparingly soluble in alcohol, and practically insoluble in chloroform and acetone. Starch was absent in the gum solution as confirmed by the iodine test for starches. The percentage ash content of the purified gum is 3.1%.  Table 2 summarizes some of the physicochemical properties of the gum.

**Figure 1:**
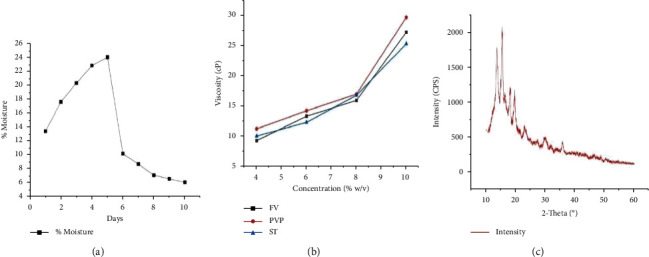
Moisture, viscosity, and crystallinity properties of *Ficus* gum. (a) Moisture sorption-desorption properties. (b) Viscosity at different concentrations at room temperature. (c) X-ray diffractogram of *Ficus* gum powder.

The moisture sorption-desorption property of *Ficus* gum is illustrated in Figure 1(a). The figure revealed that the gum has progressive moisture uptake and peak moisture loss properties depending on the environmental condition. As shown in Figure 1(b), the viscosity of the gum mucilage increased with an increase in gum concentration at room temperature. The XRD result demonstrated multiple, wide, weak peaks (without distinct sharp melting peaks) with short-range ordering, succeeded by numerous continuous halo peaks confirming its almost amorphous nature with partially crystalline fragments (Figure 1(c)). The crystallinity index of the gum powder was 45.70% which confirmed the partial crystalline nature of the amorphous powder.

#### 3.1.2. Micromeritic Properties of *Ficus* Gum

Table 3 indicates the flow-related properties of *Ficus* gum. The investigated values are all indicative of a powder that is highly compressible, freely flowing, and less cohesive [52].

#### 3.1.3. Compatibility Studies

**Figure 2:**
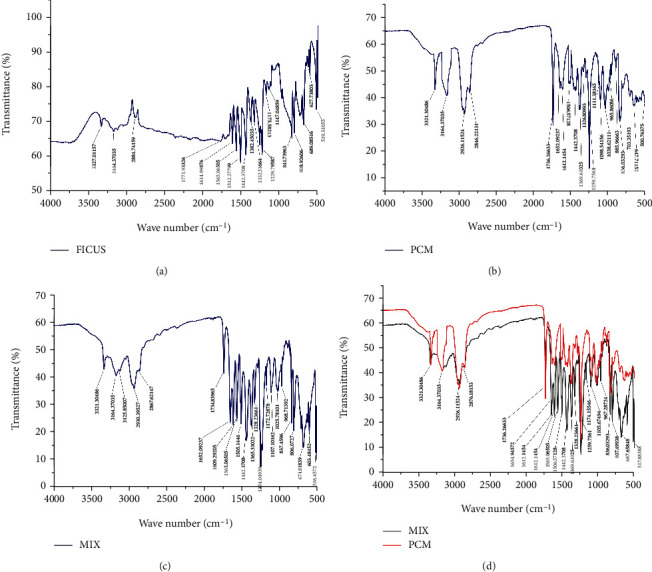
FTIR spectrums of (a) the *Ficus* gum, (b) the pure drug, (c) the physical mixture of the drug with *Ficus* gum, and (d) comparison of the pure drug vs. the mixture.

*(1) FTIR Results*. The FTIR chromatograms (Figure 2) revealed no compatibility problems between the gum and the paracetamol. The vital characteristic peaks from the FTIR spectrum of the pure drug (3321 for O-H stretching, 3164 for CH3 stretching, 3000-2846 for C-H stretching, 1652 for C=O carboxyl stretching, 1612 for C=C aromatic stretching, 1506 for C-H asymmetrical bending, 1442 for C-C stretching, 1369-1326 for C-H symmetrical bending, 1259 for C-N-H stretching, 1111 for C-O stretching, 965 for C-N (amide) stretching, and 836 for para-disubstituted aromatic ring) were also present in the spectrum of the physical mixture of the drug with the gum which ruled out the possibility of incompatibility.

**Figure 3:**
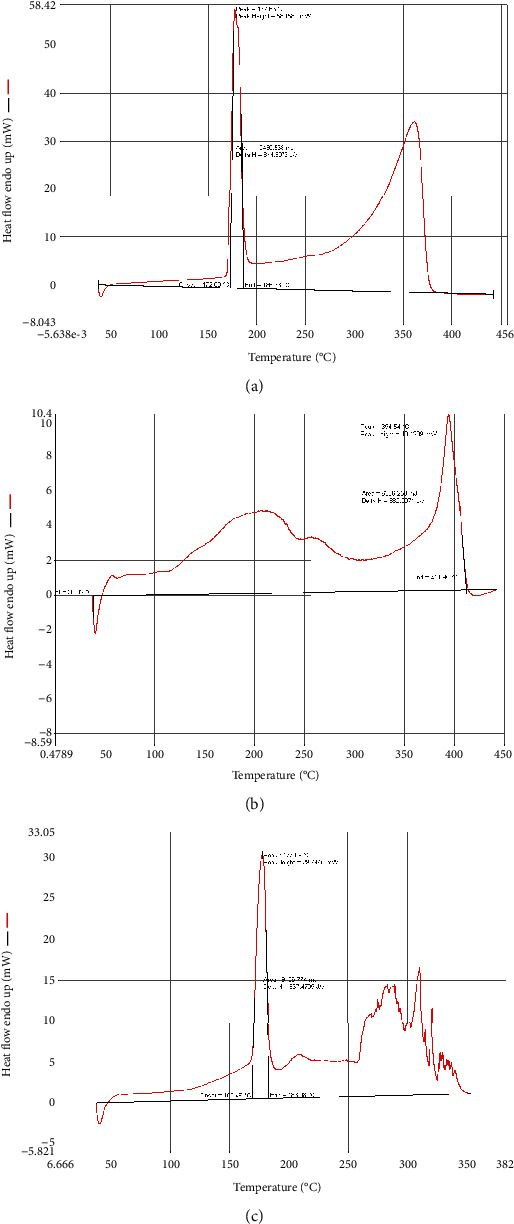
DSC thermograms of (a) the pure drug, (b) the *Ficus* gum, and (c) the drug-gum mixture.

*(2) DSC Results*. In this study, the pure drug thermogram showed a main peak and onset at 177.66° and 172.8°, respectively. In the blend, the peak and onset showed a slight shift into 177.09° and 168.49°, respectively. This confirmed that the mixture of the gum binder and the drug did not show significant changes in peak placement in comparison to the peak obtained from the pure drug, suggesting compatibility of the compounds (Figure 3).

### 3.2. Characterization of Granules

#### 3.2.1. Granule Size Properties

The mean granule size for all formulations was found to be between 479.75 *μ*m and 675.49 *μ*m, with granules of 4% w/w *Ficus* gum binder concentration and 10% w/w starch paste binder demonstrating the lower and the upper value, respectively. In addition, the percentile distribution of the granule size indicated that not less than 50% of the granules prepared with each concentration of the *Ficus* gum binder are distributed within the 315-710 *μ*m size range.

#### 3.2.2. Micromeritic Properties of Paracetamol Granules

As summarized in Table 4, the results demonstrated acceptable values of angle of repose, Carr's index, and the Hausner ratio which are below the maximum limits for good flow and compressibility. The bulk and tapped densities of the granules decreased with increasing binder concentrations. The ANOVA supported by Tukey's post hoc analysis revealed that the bulk densities of granules with *Ficus* gum binder are significantly higher than granules with respective concentrations of PVP and starch binders (*p* < 0.05). Similarly, the tapped densities of the granules with *Ficus* gum binder are significantly higher compared to granules of similar starch binder concentrations (*p* < 0.05), whereas no significant difference was observed relative to granules with PVP, except at 10% w/w concentration. There is a remarkable increase in % compressibility (represented by Carr's index) with an increase in binder concentration. Meanwhile, there was no statistically significant granule compressibility difference between the *Ficus* gum and the standard binders (*p* > 0.05), except at 10% w/w concentration where the *Ficus* gum produced granules with less compressibility behavior than that of the PVP binder (*p* < 0.05).

In this study, increasing concentrations of binders demonstrated an important decrease in the angle of repose. The angle of repose of granules with all concentration ranges of *Ficus* gum was significantly higher than those with PVP depicting lesser flow properties than PVP-based granules (*p* < 0.05), while there was comparable flow property with granules of starch binder except its better flow property at 6% w/w *Ficus* gum concentration.

#### 3.2.3. Granule Friability

In this study, the friability of the different batch granules decreased with increased binder concentration. Granules of *Ficus* gum binder demonstrated significantly higher friability than those with PVP at 4% w/w (*p* = 0.032), but no statistically significant difference in friability was observed with PVP-based granules on the other binder concentrations (*p* > 0.05). Whereas, *Ficus* gum binder resulted in less friable granules than starch binder in all binder concentrations (*p* > 0.05). Hence, *Ficus* gum has a better granulating capacity than starch while a comparable application with PVP.

### 3.3. Postcompression Tablet Quality Evaluation

#### 3.3.1. Effect of Binder Concentration on Tablet Weight, Thickness, and Diameter

There was no significant difference in the mean tablet weights of different batches except those prepared with 10% w/w PVP being significantly greater than others at similar binder concentrations (*p* < 0.05). The results did not show any statistically significant differences in tablet thickness of the test and standard binders, except at the 10% w/w concentration where tablets with the starch binder are thicker than others (*p* < 0.05). Similarly, no significant differences were observed between the diameters of the tablets (*p* > 0.05). The evaluation results for tablet weight, thickness, and diameter are presented in Table 5.

#### 3.3.2. Effect of Binder Concentration on Tablet Friability, Disintegration, and Strength

**Figure 4:**
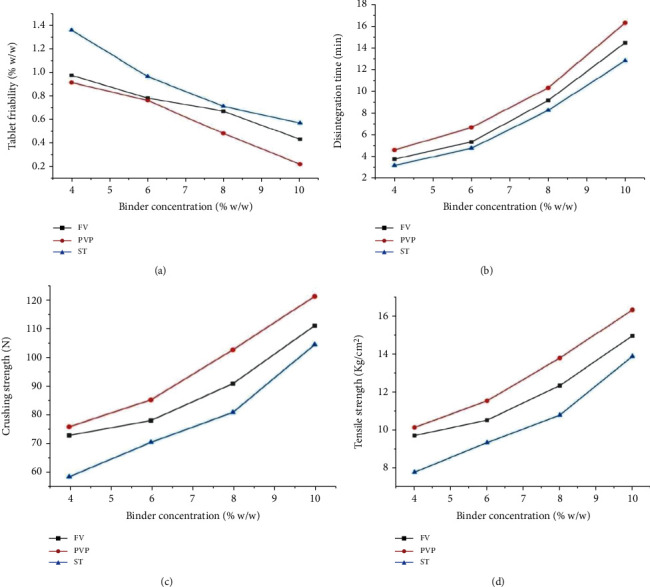
Effect of binder concentration on (a) tablet friability, (b) disintegration time, (c) crushing strength, (d) tensile strength.

Friability considerably decreased with increasing binder concentration for all batches (Figure 4(a)). Disintegration time increased with an increase in binder concentration for all batches (Figure 4(b)). The ANOVA analysis indicated that tablets formulated with different concentrations of *Ficus* gum binder demonstrated significantly higher disintegration time than that of similar concentrations of starch (*p* < 0.05), except at 6% w/w concentration. However, tablets with PVP binder needed significantly higher disintegration time than those with corresponding concentrations of *Ficus* gum and starch (*p* < 0.05). The tablet strength (both crushing and tensile) increased with increasing binder concentration for all formulations as presented in Figures 4(c) and 4(d). More specifically, tablets prepared with *Ficus* gum binder exhibited higher crushing and tensile strengths than that of all the corresponding starch binder concentrations, while they demonstrated significantly lower strength than those of PVP-based tablets (*p* < 0.05), except their insignificant difference in crushing strength at 4% w/w concentration (*p* > 0.05). Tablets with *Ficus* gum binder showed significantly higher mechanical strength than those with starch binder but lower than those with PVP binder based on the CS : FR (p < 0.05). The ranking of tablet mechanical strength based on CS : FR was generally as formulations containing PVP > *Ficus* gum > starch. The CSFR : DT revealed that tablets with *Ficus* gum binder had better quality than tablets with starch binder at every correspondent concentration and PVP-based tablets at 4 and 6% w/w concentrations (*p* < 0.05).

#### 3.3.3. *In Vitro* Drug Release Study Results

**Figure 5:**
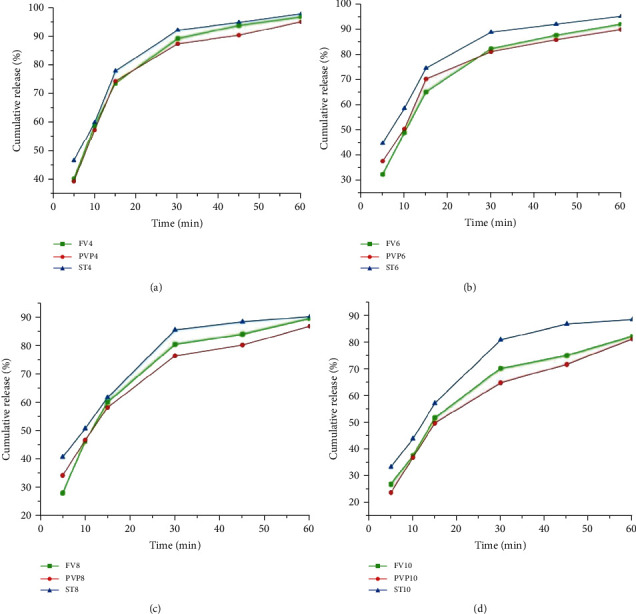
Dissolution profile of tablets (*n* = 6, mean ± SD): (a) at 4% binder conc., (b) at 6% binder conc., (c) at 8% binder conc., and (d) at 10% binder conc.

*(1) Effect of Binder Concentration on Tablet Dissolution Profile*. Tablets prepared with all concentrations, except those with 10% w/w *Ficus* gum and 8 and 10% w/w PVP binder concentrations, released 80% of the drug content in the tablet within 30 minutes which complies with pharmacopoeial standards [53]. The drug release rate of tablets generally decreased with increasing concentration of both the test and standard binders. The comparative analysis of the mean cumulative release showed that tablets with *Ficus* gum binder had a significantly lower release rate than those with similar concentrations of the starch binders (*p* < 0.05), except at 4% w/w. In contrast to this, *Ficus* gum binder resulted in a higher cumulative release than PVP binder at the corresponding concentrations (*p* < 0.05) revealing its lower hydration, less viscous film formation, or weaker bond strength formation than the PVP. Generally, as it can be observed from Figure 5, tablets with *Ficus* gum binder demonstrated an acceptable and comparable dissolution profile with the standard binders.

*(2) Effect of Binder Concentration on Drug Release Kinetics*. The regression coefficient (*R*^2^), release constant (*K*), and diffusion exponent (*n*) of the different models from the present study are presented in Table 6. The highest *R*^2^ values were observed in the first-order kinetics.

**Figure 6:**
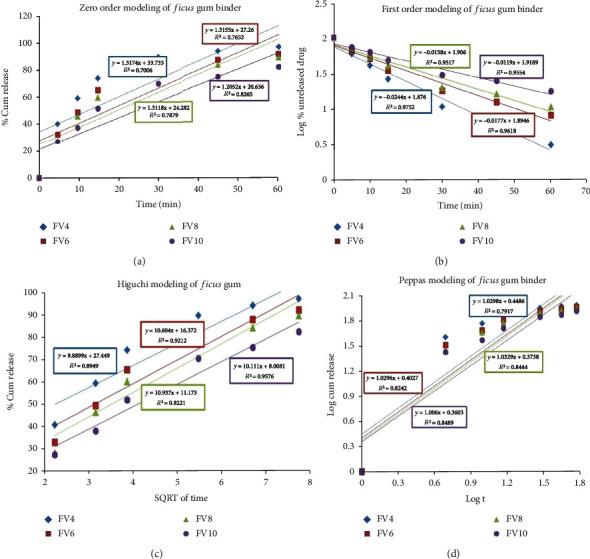
Mathematical modeling of drug release from paracetamol tablets with *Ficus* gum binder: (a) zero-order model, (b) first-order model, (c) Higuchi model, (d) Korsmeyer-Peppas model.

The formulation with 4% *Ficus* gum demonstrated the maximum fitting result (*R*^2^ = 0.9752). Additionally, all the *n* values from the Peppas model are >0.89. The different models of drug releases for the formulation are presented in Figure 6. The *t*_50_ and *t*_80_ increased while the rate constant (*K*_1_) decreased with an increase in binder concentration for both the test and the standard binders.

## 4. Discussion

### 4.1. Physicochemical Characteristics of the Gum

The percentage gum yield of *Ficus vasta* is almost similar to gum yields of other *Ficus* species such as *Ficus benjamina* (55%) [54] and *Ficus platyphylla* (52%) [47]. It is also approximated to *Ficus carica* L. in a w/v-basis yield directly from its latex [31]. *Ficus* gum has a promising yield potential compared to gum extract studies in Ethiopia such as myrrh gum (49.6%) [50] and olibanum (frankincense) (30%) [55]. It is also in the range between reported yields of gum acacia (16-68%) and Khaya and Grewia gums (30-70%) [56]. Level gum arabic yield in different varieties of *Acacia senegal* (L.) Willd in Kenya. The yield is higher in matured *Ficus* plant than the youngs. This result is in line with gum arabic yield in different varieties of *Acacia senegal* (L.) Willd in Kenya [45]. Hence, *Ficus vasta* can be considered as a good gum source. The near-neutral pH of *Ficus* gum is important since excipients with such pH properties are advantageous in the preparation of both neutral and acidic or basic drugs [57]. It has also good implications for the preparation of uncoated tablets by reducing gastrointestinal irritation. In addition, the pH obtained for this gum is consistent with the literature in that most plant gums have been reported to have acidic, neutral, or near-neutral pH [58]. Similar pH properties were also reported for guar gum [59], amada gum [52], and ghatti gum [60]. Most natural gums are water soluble with varying degrees of solubility. Moreover, gums with pharmaceutical and nutraceutical applications are often reported to have very limited alcohol and organic solubility [61]. Guar gum and gum arabic also demonstrated complete water solubility [62]. Its free solubility in the aqueous solvent may be due to the presence of branched structural features or dipole moments [63, 64]. The percentage ash content complied with the maximum value for gum acacia [46]. This is also a similar result to the study for Ethiopian gum *Acacia senegal* [65] and Kenyan gum arabic [66]. The low level of the ash value reflects its lower level of contamination and adulteration by inorganic mixtures.

The nature of the drug and excipients are determinant factors for the moisture uptake rate and hygroscopicity in tablet formulation [57]. On the contrary, the particular binding efficiency of a gum depends on its hydrophilic and viscoelastic properties [67]. The gum demonstrated a swelling index of an inherent capacity to absorb and retain the medium under different conditions to swell and break up later. This could be attributed to the presence of some hydrophilic functional groups and/or shorter chain molecules [56, 68]. The moisture content based on loss on drying (LOD) complied with the pharmacopoeial specifications (not more than 15%) [46]. This is also similar to the moisture content reports of acacia, cashew, *Albizia*, and *Khaya* gums which range between 10 and 25% [56]. The lower moisture content for *Ficus* gum suggests its stability in formulations containing moisture-sensitive drugs. The moisture sorption-desorption property of *Ficus* gum revealed that an unfortunate increase or decrease in storage temperature conditions may directly affect the hygroscopicity of the powder and formulations prepared with it as an excipient. This in turn results from higher susceptibility to microbial and physicochemical deterioration. So, storage in an air-tight container with optimum temperature conditions will be advisable to preserve the products [47].

Although the effect of different factors on viscosity varies from gum to gum, most gum dispersions will exhibit a non-Newtonian behavior most often pseudoplastic in terms of rheological behavior [69]. The increase in viscosity of the gum mucilage with an increasing concentration may be due to increased intermolecular interaction between the gum molecules and a reduction in gum-solvent interaction [63]. This is in line with other reports of gum viscosities such as myrrh gum [50] and gum of *Acacia polyacantha* [17]. Viscosity depends on the strength of attractive forces between molecules, which in turn depend on their composition, size, shape, and kinetic energy of the molecules. Therefore, any factor that can affect these elements will certainly affect viscosity [70]. Therefore, the increment in viscosity with the increasing concentration in this experiment may be due to the increase in composition with an increase in concentration, hence the increase in viscosity.

Crystal as well as a noncrystalline amorphous form may affect drug stability, dissolution rate, flow, mechanical properties, and ability to mix with excipients [48, 57]. The crystallinity nature of the gum is similar to the *Prunus domestica* exudate gum [71], gum ghatti [72], and almond gum [73]. Flow property is directly and indirectly affected by density and density-related properties of powders [57, 74]. Carr's index lower than 12% and a Hausner ratio of less than 1.2 represent a free-flowing powder. Similarly, an angle of repose less than 30° is usually indicative of good flow [75]. The investigated values are all indicative of a powder that is highly compressible, freely flowing, and less cohesive [52]. Hence, *F. vasta* gum had flowability and compressibility properties for use as a binder in tablet formulations.

### 4.2. Characterization of Granules

The drug safety, stability, and viability of the dosage form and manufacturing process can be significantly influenced by size properties. An optimal particle size with a narrower possible size distribution is required to obtain good flow properties, compaction, and hardness [50]. The mean granule values depicted that all the formulations are in line with the optimal range of mean granule size for tablets (300-1000 *μ*m) [76]. This indicates that *Ficus* gum can produce granules with optimum sizes for tableting. The results demonstrated acceptable values of angle of repose, Carr's index, and the Hausner ratio which are below the maximum limits for good flow and compressibility [74]. Hence, the test and the standard binders under this study enhanced the flow property of the drug powder mixture implying that *Ficus* gum can be used as a wet granulation tablet binder to enhance powder flow like PVP and starch. The decreased bulk and tapped densities of the granules with increasing binder concentrations indicates that the binders were capable to enlarge granules and concentrate binding bridges [77]. The difference in granule flow and compressibility properties between the different binders may be due to differences in granule density or the saturation of the binding forces at those particular binder concentration [78]. In this study, increasing concentrations of binders demonstrated an important decrease in the angle of repose. A lower angle of repose means the granules had lower interparticulate friction and uniform distribution of bridging forces and hence good flow [79]. Granule friability is mainly due to the reduction of granular size by attrition that will then affect granule strength and, consequently, the tablet quality [17]. In this study, the friability of the different batch granules decreased with increased binder concentration which may be due to the increase in bonding capacity and interparticular bridge strength attained at higher binder concentrations which in turn increases resistance against abrasion and cracking.

### 4.3. Postcompression Tablet Quality Evaluation

Weight uniformity test for tablets is a very crucial quality control test since variations in tablet weights may result in variations in drug content and overall drug bioavailability [79]. All formulations passed the weight uniformity test where not more than two tablets differed with >5% or no tablet deviated with >10% from the mean weight of each batch [80, 81]. Friability considerably decreased with increasing binder concentration for all batches which is due to the enhanced adhesive bridge formation which can resist abrasion, shock, and capping tendencies [77]. All the formulations, except those with 4% w/w starch, complied with the BP friability specifications [46]. The results showed that *Ficus* gum is capable of binding granular particles to form tablets with satisfactory properties that can withstand shock and vibrations during packaging, transportation, and use. The formulated batches also complied with the pharmacopoeial requirements for disintegration time (15 minutes) for uncoated immediate-release tablets BP [46], except those with 10% w/w PVP (17.83 ± 0.26 min). The decrease in disintegration time with increased binder concentration could be due to the reduced rate of liquid penetration into the interior of the tablets as a result of the enhanced cohesion of powder particles [67, 82].

The mechanical properties of tablets such as crushing and tensile strength, in addition to friability, are important to evaluate their ability to withstand handling during production, transportation, storage, and subsequent use. They also foretell the resistance of tablets against capping and lamination affinity, especially during the production process [67]. A tablet hardness of 4 kg F is considered to be the minimum for a satisfactory tablet based on BP specifications [83]. All tablet formulations in this study demonstrated a good hardness profile and conformed to pharmacopoeial specifications [46]. A lesser strength of tablets with *Ficus* gum compared to PVP may be due to the relatively higher bulk density of the granules with *Ficus* gum than PVP which reduces space for deformation [77]. The better strength of tablets prepared with *Ficus* gum than those with the commonly used starch binder suggests that *Ficus* gum can be used as a binder in the production of mechanically stronger tablets. Crushing strength-friability ratio (CS/FR) and CS/FR to disintegration time ratio (CS/FR : DT) can be used to evaluate the mechanical strength and the quality of the tablets in terms of strength-release balance. A higher CS/FR and CS/FR : DT ratios indicate a stronger tablet and a better balance between mechanical strength and disintegration properties, respectively [84]. The CSFR/DT, commonly known as disintegration efficiency ratio, is recommended as a better tablet quality indicator as it simultaneously evaluates the tablet's strength (crushing strength) and its weakness (friability). It also evaluates the negative effects of these parameters (tensile strength and friability) on tablet disintegration, while also assessing the usefulness of a binder in a formulation [85]. The CSFR : DT revealed that tablets with *Ficus* gum binder had better quality than tablets with starch binder at every correspondent concentration and PVP-based tablets at 4 and 6% w/w concentrations (*p* < 0.05). From these results, it can be seen that *Ficus* gum can be a promising binder with a good balance between binding and disintegration properties.

Binders and granulating agents can distinctly affect the dissolution profile of drugs [86]. In this study, tablets prepared with all concentrations, except those with 10% w/w *Ficus* gum and 8 and 10% w/w PVP binder concentrations, released 80% of the drug content in the tablet within 30 minutes which complies with pharmacopoeial standards [53]. The drug release rate of tablets generally decreased with increasing concentration of both the test and standard binders. This might be due to the higher bond strength of tablets with increased binder concentration that prolonged the dissolution time [49]. Initially, the curves are characterized by sharp slopes indicating drug release from tablets for maintaining constant plasma concentration *in vivo*. The higher dissolution rate of tablets was through the enhancement of drug solubility at lower binder concentrations. But, a lower release rate, especially at increased binder concentrations, may be due to binder hydration which leads to the formation of highly viscous and sticky films [77]. The comparative analysis implied that the higher *Ficus* gum concentration in the tablet composition formed a thicker and more viscous gel layer barrier than the starch bonding capacities preventing medium penetration into the entire tablet and as a result, retarding the drug release [87]. In contrast to this, *Ficus* gum binder resulted in a higher cumulative release than PVP binder at the corresponding concentrations (*p* < 0.05) revealing its lower hydration, less viscous film formation, or weaker bond strength formation than the PVP. This result is similar to the report on the comparative binding efficacy of almond gum [20], while it deviates from the report by Desta et al., [49] where a higher concentration of PVP binder demonstrated significantly higher cumulative release than *Acacia etbaica* Schweinf gum. Generally, as can be observed from Figure 5, tablets with *Ficus* gum binder demonstrated an acceptable and comparable dissolution profile with the standard binders. Hence, *Ficus* gum can be an alternative binder in tablet production.

Based on the *n* values of the Peppas model, the release mechanism can be Fickian or quasi-Fickian diffusion (*n* ≤ 0.45), non-Fickian diffusion (0.45 < *n* < 0.89), case II transport (*n* = 0.89), or super case II transport (*n* > 0.89) [88, 89]. Releases from some formulation systems may be classified as either pure diffusion or erosion based, while most of the systems exhibit a combination of these mechanisms [90]. As shown in Table 6, the highest *R*^2^ values were observed in the first-order kinetics. Hence, the first-order is the model that preferentially fits the formulations in this study as it best describes the drug dissolution in pharmaceutical dosage forms containing water-soluble drugs and polymers. It also represents the release from a system where the rate is concentration dependent, such that the proportion of the amount of drug released by unit time diminishes gradually [88, 91]. *Moringa oleifera* gum [92] and almond gum [20] also exhibited a first-order release kinetics from conventional paracetamol and diclofenac tablets, respectively. The formulation with 4% *Ficus* gum demonstrated the best fitting result (*R*^2^ = 0.9752). Additionally, all the *n* values from the Peppas model are >0.89, suggesting that the release mechanism from the tablets was governed by non-Fickian super case II release kinetics which is generally due to dissolution and erosion, where the drug is released as it swells, relaxes, and erodes progressively [93]. The *t*_50_ and *t*_80_ increased while the rate constant (*K*_1_) decreased with an increase in binder concentration for both the test and the standard binders. This implies that the drug was dissolving at a faster rate as time goes. It would appear that changes in the surface area of the dissolving particles brought about by the disintegration and deaggregation of the tablets were manifested in the substantial increase in dissolution rate with increasing time. Similar results were observed from the study on the binding effect of *Eucalyptus tereticornis* gum [94].

## 5. Conclusion

This study demonstrated the good binding ability of *Ficus* gum as a pharmaceutical binder in tablet formulation. Extraction of the gum provided a considerable yield of purified gum. The gum was also found to be water soluble and exhibited excellent flowability and compressibility with no incompatibility problem. The paracetamol granules prepared by wet granulation technique using the *Ficus* gum as a binder demonstrated adequate flow and compression properties comparable to those prepared with the standard binders. The tablets prepared with *Ficus* gum also exhibited acceptable and comparable physical, mechanical, and drug release properties. Especially, tablets demonstrated better quality profiles at 4 and 6% w/w *Ficus* gum binder concentrations. Furthermore, the gum uniquely demonstrated desirable characteristics in terms of yield, water solubility, micromeritic property, and compatibility which are rarely attained together from a single gum. Therefore, it can be concluded from this study that the gum of *Ficus vasta* Forssk can be a possible alternative excipient as a binder in granule and tablet formulations. The comparison was made in the working concentration of the standard binders in immediate release tablets. Hence, the result indicates that *Ficus* gum can be a good candidate for immediate release tablet manufacturing. However, the increase in binding efficacy with increase in gum concentration may indicate its applicability in modified release formulations. This may be an important clue for further optimization of its use as a tablet binder for industrial applications in the manufacturing of dosage forms with various drug release characteristics.

## Figures and Tables

**Table 1:** Composition of the different batches of paracetamol tablets.

Ingredients	Composition per individual tablet of a batch in mg											
	FV4	FV6	FV8	FV10	PVP4	PVP6	PVP8	PVP10	ST4	ST6	ST8	ST10
Paracetamol API	250	250	250	250	250	250	250	250	250	250	250	250
*Ficus* gum	14.4	21.6	28.8	36.0	—	—	—	—	—	—	—	—
PVP–K30	—	—	—	—	14.4	21.6	28.8	36.0	—	—	—	—
Starch^@^1500	—	—	—	—	—	—	—	—	14.4	21.6	28.8	36.0
Starch disintegrant	36	36	36	36	36	36	36	36	36	36	36	36
Mg stearate	3.6	3.6	3.6	3.6	3.6	3.6	3.6	3.6	3.6	3.6	3.6	3.6
Talc	1.8	1.8	1.8	1.8	1.8	1.8	1.8	1.8	1.8	1.8	1.8	1.8
Lactose	54.2	47	39.8	32.6	54.2	47	39.8	32.6	54.2	47	39.8	32.6
Total wt. (mg/tab)	**360**	**360**	**360**	**360**	**360**	**360**	**360**	**360**	**360**	**360**	**360**	**360**

**Table 2:** Physicochemical properties of the FV gum.

Parameters	Determined value^∗^
%Yield	
Younger tree (%w/w)	38.46 ± 0.85
Old tree (%w/w)	50.63 ± 1.15
Latex (%w/v)	33.92 ± 1.63
pH	7.62 ± 0.025
Total ash (%)	3.10 ± 0.250
Swelling capacity	2.93 ± 0.29
Loss on drying (%)	10.97 ± 0.235
Starch	Negative
Water solubility index (%)	62.6 ± 1.098
%Solubility (w/v)	
Cold water	10
Hot water	10
Ethanol	0.79 ± 0.001
Chloroform	0.02
Acetone	0.04

^∗^mean ± SD: (*n* = 3).

**Table 3:** Micromeritic properties of the *Ficus* gum.

Characteristics	Determined value^∗^
Bulk density (g/ml)	0.693 ± 0.006
Tapped density (g/ml)	0.763 ± 0.005
Carr's index (%)	9.133 ± 0.192
Hausner ratio	1.101 ± 0.002
Flow rate (g/sec)	16.55 ± 1.29
The angle of repose (Ө)	22.01 ± 0.592

^∗^mean ± SD: (*n* = 3).

**Table 4:** Summary of micromeritics properties of paracetamol granule, mean ± SD: (*n* = 3).

Batch	Bulk density	Tapped density	Carr's index (%)	Hausner ratio	Flow rate (g/sec)	Angle of repose
FV4	0.594 ± 0.007	0.620 ± 0.018	4.2 ± 0.017	1.044 ± 0.018	12.757 ± 0.790	29.757 ± 0.791
FV6	0.555 ± 0.006	0.585 ± 0.011	5.1 ± 0.008	1.053 ± 0.009	13.257 ± 0.756	29.093 ± 0.111
FV8	0.527 ± 0.008	0.559 ± 0.016	5.7 ± 0.012	1.059 ± 0.014	13.397 ± 0.796	28.467 ± 0.497
FV10	0.501 ± 0.003	0.539 ± 0.003	7.0 ± 0.005	1.075 ± 0.006	13.287 ± 1.121	27.737 ± 0.431
PVP4	0.580 ± 0.004	0.614 ± 0.005	5.5 ± 0.003	1.058 ± 0.004	13.103 ± 0.110	27.287 ± 0.437
PVP6	0.545 ± 0.004	0.579 ± 0.002	5.9 ± 0.006	1.062 ± 0.006	14.497 ± 0.501	26.987 ± 0.196
PVP8	0.506 ± 0.003	0.539 ± 0.003	6.2 ± 0.001	1.066 ± 0.001	14.487 ± 0.522	26.440 ± 0.769
PVP10	0.453 ± 0.006	0.485 ± 0.003	6.7 ± 0.007	1.072 ± 0.008	14.803 ± 1.069	25.527 ± 0.376
ST4	0.481 ± 0.002	0.510 ± 0.003	5.7 ± 0.003	1.060 ± 0.003	10.190 ± 1.037	31.083 ± 0.225
ST6	0.460 ± 0.001	0.491 ± 0.002	6.2 ± 0.001	1.066 ± 0.001	10.253 ± 1.176	30.523 ± 0.420
ST8	0.450 ± 0.002	0.482 ± 0.002	6.5 ± 0.001	1.069 ± 0.001	10.607 ± 0.653	29.393 ± 0.350
ST10	0.438 ± 0.003	0.472 ± 0.003	7.2 ± 0.001	1.077 ± 0.001	11.257 ± 0.965	28.327 ± 0.342

**Table 5:** Summary of paracetamol tablet weight, thickness, and diameter properties.

Batch	Weight^a^ (mg)	Diameter (mm)^b^	Thickness (mm)^b^
FV4	362.45 ± 5.71	9.638 ± 0.013	5.088 ± 0.073
FV6	357.45 ± 8.25	9.646 ± 0.042	5.028 ± 0.088
FV8	360.70 ± 6.13	9.632 ± 0.015	5.004 ± 0.087
FV10	356.50 ± 7.30	9.628 ± 0.022	5.028 ± 0.051
PVP4	359.05 ± 6.55	9.628 ± 0.015	5.082 ± 0.043
PVP6	358.15 ± 7.22	9.644 ± 0.017	5.030 ± 0.039
PVP8	362.60 ± 6.80	9.636 ± 0.018	5.064 ± 0.042
PVP10	365.45 ± 4.75	9.630 ± 0.016	5.030 ± 0.032
ST4	363.10 ± 7.42	9.648 ± 0.018	5.092 ± 0.048
ST6	363.05 ± 5.96	9.636 ± 0.011	5.114 ± 0.036
ST8	359.10 ± 4.52	9.632 ± 0.013	5.088 ± 0.051
ST10	357.50 ± 7.50	9.626 ± 0.018	5.106 ± 0.023

mean ± SD; ^a^*n* = 20; ^b^*n* = 5.

**Table 6:** Characteristics of the kinetic models.

Batch	Zero order		First order		Higuchi		Korsmeyer-Peppas		
	*K*_0_	*R* ^2^	*K*_1_	*R* ^2^	*K*_*H*_	*R* ^2^	*K*_*P*_	*R* ^2^	*N*
FV4	1.317	0.7006	0.056	0.975	9.890	0.8949	2.809	0.7917	1.03
FV6	1.312	0.7652	0.041	0.962	10.60	0.9212	2.528	0.8242	1.03
FV8	1.312	0.7879	0.036	0.952	10.94	0.9221	2.365	0.8444	1.03
FV10	1.205	0.8265	0.027	0.955	10.11	0.9576	2.293	0.8489	1.00
PVP4	1.277	0.6884	0.047	0.948	9.514	0.8772	2.604	0.7903	1.02
PVP6	1.220	0.7078	0.036	0.920	9.222	0.8903	2.719	0.7962	1.01
PVP8	1.208	0.7743	0.031	0.947	9.524	0.9521	2.563	0.8140	1.00
PVP10	1.185	0.8485	0.026	0.969	10.06	0.9713	2.204	0.8601	1.00
ST4	1.288	0.6640	0.062	0.971	9.192	0.8822	2.971	0.7723	1.02
ST6	1.251	0.6725	0.048	0.942	8.969	0.8949	2.928	0.7748	1.01
ST8	1.263	0.7418	0.039	0.916	9.670	0.9259	2.720	0.7973	1.01
ST10	1.303	0.7925	0.037	0.939	10.67	0.9374	2.484	0.8283	1.02

## Data Availability

The experimental data used to support the findings of this study are available from the corresponding author upon request.

## Abbreviations

ANOVA: Analysis of variance
API: Active pharmaceutical ingredient
BP: British Pharmacopeia
CS/FR: Crushing strength-friability ratio
DC: Direct compression
DSC: Differential scanning calorimetry
DT: Disintegration time
FTIR: Fourier transform infrared spectroscopy
EMC: Equilibrium moisture content
FV: *Ficus vasta*
GMP: Good manufacturing practice
HPMC: Hydroxy propyl methyl cellulose
IP: Indian Pharmacopoeia
GRAS: Generally recognized as safe
LOD: Loss on drying
MUR: Moisture uptake rate
PEG: Polyethylene glycol
PVC: Polyvinyl chloride
PVP: Polyvinylpyrrolidone
SP: Swelling power
ST: Starch
USP: United States Pharmacopeia
UV/Vis: Ultraviolet-visible spectroscopy
WSI: Water solubility index
XRD: X-ray diffraction.
